# Validity and reliability of the ERSA questionnaire in Turkish

**DOI:** 10.1093/deafed/enad064

**Published:** 2024-01-12

**Authors:** Hüseyin Öztürk, Mustafa Karabulut, Mine Baydan-Aran, Suna Tokgöz-Yılmaz

**Affiliations:** Audiology Balance and Speech Disorders Unit, Department of Otorhinolaryngology, Faculty of Medicine, Ankara University, Ankara, Türkiye; Division of Balance Disorders, Department of Otorhinolaryngology and Head and Neck Surgery, School for Mental Health and Neuroscience, Maastricht University Medical Center, Maastricht, The Netherlands; Audiology Balance and Speech Disorders Unit, Department of Otorhinolaryngology, Faculty of Medicine, Ankara University, Ankara, Türkiye; Audiology Balance and Speech Disorders Unit, Department of Otorhinolaryngology, Faculty of Medicine, Ankara University, Ankara, Türkiye

## Abstract

This methodological study aimed to assess the validity and reliability of the Turkish version of the Evaluation of the Impact of Hearing Loss in Adults (ERSA) questionnaire for individuals with treated hearing loss. The study involved 200 participants, and both exploratory factor analysis and confirmatory factor analysis were used to examine structural validity. External validity was assessed by correlating ERSA scores with the Abbreviated Profile of Hearing Aid Benefit (APHAB). Internal consistency and test–retest reliability were evaluated using Cronbach’s alpha and the intraclass correlation coefficient, respectively. The Turkish ERSA demonstrated strong psychometric properties, with significant correlations between APHAB and ERSA scores and excellent internal consistency and test–retest reliability. The findings suggest that the Turkish ERSA is a valid and reliable tool for evaluating the impact of hearing loss in individuals.

## The impact of hearing loss: prevalence, trends, and consequences

Hearing loss has emerged as a prominent global disability, ranking as the third most prevalent cause of disability worldwide (Haile et al., 2021). According to the World Health Organization, more than 1.5 billion individuals, accounting for ~20% of the global population, are affected by hearing loss. There is a growing incidence of hearing loss, indicating that this number will reach ~2.5 billion by the year 2050 (Organization, 2022). According to 2016 data in Türkiye , the prevalence of hearing problems stands at 6.9% across all age groups, with rates of 5.3% for men and 8.6% for women. The prevalence varies further with 2.1% for individuals aged 15–44, 9.4% for those aged 45–54, 13.3% for the 55–64 age group, 18.5% for individuals aged 65–74, and 31.9% for those aged over 75 (General Directorate of Services for Persons With Disabilities and the Elderly Disability and Ageing Statistical Bulletin, 2020). This pervasive issue can have long-term consequences for individuals, including social isolation, stigma, psychological distress, depression, strained family relationships, limited career opportunities, and professional stress (Davis & Hoffman, 2019; Huddle et al., 2017; Nordvik et al., 2018; Olusanya et al., 2014). Consequently, hearing loss can have a significant negative effect on quality of life.

To comprehensively evaluate the impact of hearing loss on an individual’s quality of life, it is essential to use both objective and subjective approaches. Objective tests widely used in clinical settings, including otoacoustic emissions, immittance measurements, electrocochleography, and auditory brainstem responses, are well-established and reliable methods (Hall & Swanepoel, 2009); however, these tests tend to be time consuming. Furthermore, while objective measurements provide quantifiable data about an individual’s hearing sensitivity and can identify specific hearing disorders or abnormalities, these tests are typically conducted in controlled and quiet environments, which do not mimic the complexity of real-world listening situations. In contrast, subjective assessment approaches, such as patient-reported outcome measures (PROMs), offer a simpler alternative and enable the collection of substantial data within a short timeframe and involve self-reporting by individuals about their hearing experiences in daily life. These assessments take into account factors like background noise, communication challenges, and the impact of hearing difficulties on one’s quality of life. (Ramakers et al., 2017). These measures have gained increasing significance in contemporary healthcare services as they provide valuable insights into the impact of hearing loss on individuals’ quality of life (Ciorba et al., 2012).

## Patient-reported outcome measures

PROMs are self-reported assessments that capture information directly from patients about their health status, symptoms, functioning, well-being, and quality of life. They provide insights into how patients perceive and experience their condition or treatment (Churruca et al., 2021). The literature highlights several PROMs that are utilized to assess the quality of life in individuals with hearing loss. In Türkiye, the commonly used PROMs include the Abbreviated Profile of Hearing Aid Benefit (APHAB) (Cox & Alexander, 1995), the Nijmegen Cochlear Implant Questionnaire (NCIQ) (Hinderink et al., 2000), and the Glasgow Benefit Inventory (GBI) (Kubba et al., 2004). These PROMs are designed for individuals with treated hearing loss and offer a convenient means of capturing subjective perspectives on symptoms, functional status, experience, satisfaction, and overall quality of life. However, it is important to acknowledge that these PROMs have certain limitations. For instance, the GBI assesses changes in quality of life compared to a previous situation but does not provide an independent evaluation at a specific time point (e.g., pre-amplification period) (Kubba et al., 2004). The NCIQ, although valuable for measuring improvement or deterioration, has demonstrated reliability issues in self-esteem and speech production subdomains (Hinderink et al., 2000). The APHAB measures communication difficulties in typical situations but often faces incomplete responses due to question phrasing and the complexity of its scoring system. Moreover, certain questions may refer to unfamiliar conditions, leading to skipped responses (Cox & Alexander, 1995). In addition, when considering a more comprehensive viewpoint, individuals have the capacity to either underestimate or overestimate their experiences, and in some cases, they may refrain from sharing certain experiences due to feelings of embarrassment or the weight of social stigma. Moreover, the presence of an interviewer or close relatives can influence respondents to provide responses that align with social desirability, potentially skewing the accuracy and depth of their self-reported information. Given these limitations, there is a clear need for a comprehensive and user-friendly questionnaire that can efficiently and realistically evaluate the impact of hearing loss on quality of life.

Considering the aforementioned circumstances, Ambert-Dahan et al. developed the Evaluation of the Impact of Hearing Loss in Adults: Évaluation du Retentissement de la Surdité chez l’Adulte (ERSA) questionnaire (Ambert-Dahan et al., 2018). They developed this questionnaire with French-speaking individuals who sought consultation for hearing loss like potential candidates for cochlear implantation or had already received a cochlear implant. They conducted assessments to evaluate content validity (ensuring that the selected questions comprehensively covered all aspects of the measured concept), structural or internal validity (evaluating the coherence of the scale’s internal organization), external validity, internal coherence, and the reproducibility of quality-of-life scores during repeated measurements. This self-reported questionnaire is designed to assess the impact of hearing loss on daily activities and can be completed in ~5 min. It comprises questions that are easily comprehensible to patients and allows for an evaluation of their current experience rather than their previous state. Given the prevalence of hearing loss and the need for a practical PROM for individuals with treated hearing loss in the Turkish population, it is crucial to utilize this questionnaire in Turkish. Therefore, the aim of this study is to examine the validity and reliability of the Turkish version of ERSA questionnaire among individuals with treated hearing loss.

## Method

### Ethical consideration

Written permission was obtained from the authors of the original ERSA questionnaire, developed at the “AP–HP, Groupe Hospitalier Pitié-Salpêtrière, Service d’ORL, Otologie, Implants Auditifs et Chirurgie de la Base du Crâne, Boulevard de l’Hôpital” center, to use and adapt the questionnaire for the current study. The research protocol was approved by the ethics committee of Ankara University (decision no: 2022-368, date: 09.03.2022). All participants in the study provided written informed consent prior to their participation.

### Translation and cultural adaptation

An established guideline on how to translate a questionnaire was used during the translation process (Tsang et al., 2017). The process of translating the original English version of the questionnaire into Turkish involved two independent translators, one specializing in audiology and the other in sociology, both of whom were bilingual native Turkish speakers. The translations provided by the two translators were combined to create the Turkish version of the questionnaire. Once the translators completed the translation process, a consensus meeting was organized to compare their work and address any disagreements or challenges they encountered. During this collaborative effort, two key issues were identified: language complexity and contextual differences, which had an impact on two of the translators. To ensure standardization, these challenges were resolved by seeking consultation from another independent translator. Subsequently, cultural adaptations were discussed and incorporated into the questionnaire. Adapting certain terminology to align with Turkish conditions was considered essential. For instance, changing “acquaintances” to “knowledge” and “friends and family” to “your loved ones” improved item comprehension significantly. Following the initial translation, the Turkish version underwent a back-translation process by a different specialist who was not involved in the initial translation. A comparison was made between the back-translated questionnaire and the original version to ensure that the meanings of the components remained consistent. During this comparison, some discrepancies in word choices between the original ERSA version and the back-translated ERSA version were identified. It is important to note, however, that these discrepancies did not affect the overall meaning of the items. Once the necessary checks were completed, the Turkish version of the questionnaire was administered to the study participants.

In our study, we ensured that the methods used to assess the validity and reliability of the ERSA questionnaire adhered to established standards and practices in audiology (Gündüz et al., 2021; Özses et al., 2022; Yanik et al., 2008). We followed well-established protocol and guideline that are commonly employed in similar research within the field of audiology (Hall et al., 2018). There were no departures from these established standards, nor did we employ any innovative or unconventional approaches in our methodology.

### Participants

In the literature, various recommendations are provided regarding the optimal sample size for conducting factor analysis. The minimum sample sizes suggested range from 3 to 20 times the number of variables, with absolute ranges varying from 100 to over 1,000 participants (Mundfrom et al., 2005). It is commonly advised that each questionnaire item should have a minimum of 10 participants, establishing an ideal participant-to-item ratio of 10:1 (Nunnally, 1978). Considering these guidelines and the fact that the ERSA questionnaire comprises 20 items, a total of 200 participants were required for this study.

Eligible participants were individuals aged 18 years and older with from mild acquired to profound acquired hearing loss, experiencing hearing loss complaints in at least one ear for a minimum duration of 6 months, without any psychiatric disorders, and native Turkish speakers. Participants with all types of hearing loss were included, while those who had not previously received treatment for hearing loss (e.g., medical treatment, hearing amplification, surgery) were excluded. Demographic information of all participants was collected, and both the ERSA and APHAB questionnaires were completed by all participants. The inclusion of APHAB was based on its frequent use in the literature for assessing the impact of hearing loss on quality of life. After a 1-month interval, the participants completed the ERSA questionnaire for a second time to evaluate test–retest reliability.

### Outcome measures

*The Evaluation of the Impact of Hearing Loss in Adults: Évaluation du Retentissement de la Surdité chez l'Adulte (ERSA)* questionnaire is a self-reported measure consisting of 20 items. Its purpose is to assess the impact of hearing loss on various aspects of daily-life activities. The questionnaire is divided into four sub-categories, namely quality of life, personal life, social life, and professional life. These components allow participants to provide responses based on their current sentiments, without referring to previous circumstances. Each sub-category employs a visual analog questionnaire, where scores of 0, 5, or 10 points are assigned to three statements that capture the essence of the question being asked. For example, response options may include “none/little” or “a lot/completely,” or “never/sometimes/generally.” There are no reverse-scored items in any of the components. A score of 0 always indicates the most challenging situation, while a score of 10 represents the optimal situation. Consequently, lower scores indicate a greater negative impact of hearing loss on quality of life. The maximum achievable score is 200 (ERSA/200) for individuals who are employed, and 150 (ERSA/150) for those who do not have a profession or are retired. The ERSA/200 and ERSA/150 versions differ only in the inclusion of the professional life sub-category (Ambert-Dahan et al., 2018).

*The Abbreviated Profile of Hearing Aid Benefit questionnaire (APHAB)*, developed by Cox and Alexander in 1995, is a self-assessment measure consisting of 24 items. It is designed to assess communication difficulties experienced in everyday life situations and is divided into four sub-categories: ease of communication, reverberation, background noise, and aversiveness. The questions are presented in a random order and may occasionally involve reverse scoring. Participants are asked to rate their experience with each item using one of seven response options, ranging from “always” to “never.” The questionnaire is scored on a scale from 0 to 100%, where 0 indicates no difficulties and 100% represents the maximum negative impact of hearing loss on quality of life (Cox & Alexander, 1995).

### Procedure

Pure tone audiometry and immittance measurements were conducted to verify the hearing status of the participants. Air conduction pure tone audiometry was performed using the Interacoustics AC-40 clinical audiometer (Interacoustics, Denmark), covering a frequency range of .25–8 kHz. Bone conduction evaluation was carried out using the Radioear B-71 bone conduction vibrator (Radioear, USA), with a frequency range of .5–4 kHz. Immittance measurements included tympanometry and acoustic reflex measurements. The ASHA (American Speech-Language-Hearing Association) guideline was utilized to determine the degree of hearing loss, while the air–bone gap was considered to identify the type of hearing loss. Moreover, the immittance measurements were cross-validated with the findings from pure tone audiometry.

The ERSA and APHAB questionnaires were administered to patients either in person or via email. Clear instructions were provided to the patients on how to complete the questionnaires, for example, indicating the scale for responses (e.g., “0: Not at all, 10: Totally” for the question “Do you have a satisfactory social life despite your hearing loss?”). In cases where patients were illiterate, their relatives were contacted, and the questions were answered through interviews. For participants who were able to read and respond independently, they completed the questionnaires themselves.

### Statistical analysis

The data analysis was conducted using Statistical Package for the Social Sciences (SPSS) version 21.0 and Analysis of Moment Structures (AMOS) version 21.0 software. The psychometric properties of the ERSA were assessed in terms of validity and reliability. The normality of the data distribution was assessed using the Shapiro–Wilk test to determine if the data followed a normal distribution (Razali & Wah, 2011).

To evaluate the validity of the ERSA questionnaire, both structural validity and external validity were examined. Structural validity was assessed using two methods: exploratory factor analysis (EFA) and confirmatory factor analysis (CFA). EFA was employed to analyze the factor loadings of the ERSA and its sub-categories. In CFA, the significance of the model fit was determined based on the root mean square error of approximation (RMSEA), with values below 8% considered significant (Alpar, 2013; McDonald & Marsh, 1990). Furthermore, additional fit indices, including the comparative fit index (CFI) and the Chi-square/degree of freedom ratio (*χ*^2^/df), were calculated. A CFI value above .9 (Cicchetti, 1994) and a *χ*^2^/df ratio below 5 were considered statistically significant (Carmines, 1983). To examine the external validity, the Pearson correlation coefficient between the ERSA and APHAB questionnaires was computed.

To evaluate the reliability of the ERSA questionnaire, both internal consistency and test–retest analyses were conducted. Internal consistency was assessed using Cronbach’s alpha, with the following interpretation: not reliable (<.40), low reliability (.40–.60), moderate reliability (.60–.80), or high reliability (.80–1.0) (Cronbach et al., 1965). For the test–retest analysis, participants completed the Turkish version of ERSA twice: once on the day of admission and again 1 month after admission. Test–retest reliability was measured using the intraclass correlation coefficient (ICC), with the following interpretation: weak (<.40), moderate (.40–.60), good (.60–.75), or excellent (.75–1.00) (Alpar, 2013). Statistical significance was set at *p* < .05.

## Results

The study enrolled a total of 200 participants, comprising 103 males (52%) and 97 females (48%). The mean age was 41.61 ± 14.71 years, ranging from 18 to 60 years. Among the participants, 39.5% had completed high school, 34% had completed primary school, 21% were university graduates, and 5.5% were illiterate. The distribution of hearing loss types showed that 70% had sensorineural hearing loss, 17% had mixed-type hearing loss, and 13% had conductive hearing loss. In terms of the degree of hearing loss, 24.5% had mild, 22.5% had moderate-to-severe, 22% had moderate, 16% had severe, and 15% had severe-to-profound hearing loss. Bilateral hearing loss was observed in 82.5% of the participants, while 17.5% had unilateral hearing loss. Table 1 presents the demographic characteristics of the study participants.

**Table 1:** Demographic information of the study participants.

Variables	Groups	Number (N)	Percentage (%)
Sex	Female	97	48.5
	Male	103	51.5
Age (years)	Mean ± *SD*: 41.61 ± 14.71 Range: 18–60		
Profession	Employed	86	43
	Unemployed	114	57
Education level	Illiterate	11	5.5
	Primary school	68	34
	High school	79	39.5
	University	42	21
Type of hearing loss	CHL	26	13
	SNHL	140	70
	Mixed	34	17
Degree of hearing loss	Mild	49	24.5
	Moderate	44	22
	Moderate-to-severe	45	22.5
	Severe	32	16
	Severe-to-profound	30	15
Side of hearing loss	Unilateral	35	17.5
	Bilateral	165	82.5

*Note. SD* = standard deviation; CHL = conductive hearing loss; SNHL = sensorineural hearing loss.

The Kaiser–Meyer–Olkin (KMO) measure of sampling adequacy yielded values of ≥.8 for all factors, indicating that the sample size was suitable for conducting factor analysis. The Bartlett’s test of sphericity showed *p* values of <.001, indicating that the data met the assumptions necessary for factor analysis. EFA of the questionnaire revealed the presence of four distinct factors, with factor loadings ranging from .716 to .867. To further assess the accuracy of the four factors identified in the EFA, CFA was conducted. The fit indices, including the RMSEA, comparative fit index, and Chi-square/degree of freedom ratio, were analyzed. The results indicated that the fit indices for all sub-categories were within acceptable values (see Table 2).

**Table 2:** Explanatory factor analysis and the confirmatory factor analysis of the ERSA.

	Exploratory factor analysis					Confirmatory factor analysis		
						Acceptable fit indexes		
Sub-categories	Item	Factor load	Explained variance ratio	Kaiser–Meyer–Olkin Measure of Sampling Adequacy	Bartlett’s test of sphericity (*p* value)	Chi-square/degree of freedom ratio < 5	Comparative fit index > .90	Root mean square error of approximation < .08
Quality of life	QL1	.815						
	QL2	.809						
	QL4	.789	55.318	.804	.000	.746	1.000	.000
	QL3	.761						
	QL5	.495						
Personal life	PL3	.812						
	PL1	.772						
	PL2	.771	57.604	.824	.000	2.163	.981	.076
	PL5	.719						
	PL4	.716						
Social life	SL2	.867						
	SL1	.827						
	SL4	.809	65.700	.849	.000	1.890	.992	.067
	SL3	.777						
	SL5	.769						
Professional life	PRL1	.856						
	PRL4	.849						
	PRL2	.827	66.757	.857	.000	1.145	.996	.041
	PRL3	.824						
	PRL5	.722						

*p < .05: Pearson correlation test*

*Note.* QL = quality of life; PL = personal life; SL = social life; PRL = professional life.

Significant moderate to strong correlations were observed between the sub-categories of the APHAB and the sub-categories of the ERSA, with the exception of the aversiveness sub-category. The correlation coefficients (*r*) ranged from .404 to .622, all of which were statistically significant (*p* < .05). The highest positive correlation was observed between the ERSA professional life sub-category and the APHAB total score (*r* = .622). On the other hand, the lowest correlation was found between the APHAB total score and the ERSA quality of life sub-category (*r* = .404). No significant correlation was found between the ERSA and the aversiveness sub-category of the APHAB (*p* > .05). These findings are presented in Table 3.

**Table 3:** External validity analysis between the ERSA and ABHAP.

ERSA												
	Quality of life		Personal life		Social life		Professional life		ERSA/150 total score		ERSA/200 total score	
	*r*	*p*	*r*	*p*	*r*	*p*	*r*	*p*	*r*	*p*	*r*	*p*
*APHAB*												
Ease of communication	.463	**<.001**	.531	**<.001**	.547	**<.001**	.610	**<.001**	.567	**<.001**	.516	**<.001**
Background noise	.421	**<.001**	.466	**<.001**	.536	**<.001**	.519	**<.001**	.525	**<.001**	.513	**<.001**
Reverberation	.462	**<.001**	.430	**<.001**	.428	**<.001**	.509	**<.001**	.449	**<.001**	.455	**<.001**
Aversiveness	−.016	.819	.065	.357	.120	.092	.218	.054	.067	.345	.172	.114
APHAB total score	.404	**<.001**	.496	**<.001**	.545	**<.001**	.622	**<.001**	.534	**<.001**	.590	**<.001**

*Note.* APHAB = Abbreviated Profile of Hearing Aid Benefit questionnaire; ERSA = Evaluation of the Impact of Hearing Loss in Adults; *r* = correlation coefficient. *p*<0.05.

The ERSA questionnaire demonstrated moderate to excellent internal consistency across both total scores and sub-category scores. The Cronbach’s alpha coefficients for the ERSA/200 total score and ERSA/150 total score were .94 and .92, respectively. The sub-categories of quality of life, personal life, social life, and professional life exhibited internal consistency with Cronbach’s alpha values of .78, .81, .86, and .87, respectively. The quality of life sub-category demonstrated a moderate level of internal consistency with a Cronbach’s alpha of .78. Removal of any individual item from the questionnaire did not significantly impact the internal consistency coefficients, as all remaining items maintained values above the acceptable threshold (>.80). These results are presented in Table 4.

**Table 4:** Internal consistency and test–retest of the ERSA and sub-categories.

Internal consistency analysis test–retest reliability analysis				
	Cronbach’s alpha (*N*:200)	ICC (*N*:50)	ICC (95% CI)	*p*
Quality of life	.78	.90	.85–.93	.000
Personal life	.81	.86	.80–.91	.000
Social life	.86	.91	.86–.94	.000
ERSA/150 total score	.92	.94	.90–.97	.000
	Cronbach’s alpha (*N*:86)	ICC (*N*:24)	ICC (95% CI)	*p*
Professional life	.87	.92	.87–.96	.000
ERSA/200 total score	.94	.95	.89–.98	.000

*Note.* ICC = intraclass correlation coefficient; CI = confidence interval; *N* = number.

The test–retest reliability of the ERSA/150 and ERSA/200 total scores demonstrated excellent agreement, with ICCs of .94 (95% CI: .90–.97) and .95 (95% CI: .89–.98), respectively. The sub-categories also exhibited strong test–retest reliability, with ICC values of .90 (95% CI: .85–.93) for quality of life, .86 (95% CI: .80–.91) for personal life, .91 (95% CI: .86–.94) for social life, and .92 (95% CI, .87–.96) for professional life. All correlations between the test and retest administrations were statistically significant (*p* < .001). These findings indicate a high level of repeatability and consistency of the questionnaire across total scores and sub-categories. Detailed results are presented in Table 4.

## Discussion

The process of developing quality of life questionnaires is known to be time consuming, but the adaptation of such questionnaires to different languages and cultures has become increasingly common, aiming to establish a global standard. However, it is evident from the literature that there is no single questionnaire that comprehensively assesses all aspects of the impact of hearing loss. While several existing questionnaires in the literature can provide a quick assessment of the impact on quality of life, they may also have certain limitations. Therefore, there is a need for a straightforward, rapid, and practical approach to evaluating the impact of hearing loss (Ambert-Dahan et al., 2018).

The present study aimed to evaluate the psychometric properties of the ERSA questionnaire in individuals with treated hearing loss. The findings of the study revealed that the Turkish version of the ERSA exhibited moderate to strong measurement properties. Consequently, it can be concluded that the ERSA is a valid and reliable assessment tool suitable for both research purposes and practical applications in individuals with treated hearing loss.

EFA and CFA were conducted to assess the structural validity of the ERSA questionnaire. EFA examined three key aspects: the KMO measure, Bartlett’s test of sphericity, and factor loadings. Firstly, the KMO values were examined to determine the adequacy of the sample size. Values above .8 indicated sufficient sample size, while values between .7 and .79 indicated a moderate level, and values between .6 and .69 indicated insufficiency (Shrestha, 2021). In the present study, KMO values above .8 were obtained for all ERSA sub-categories, indicating sufficient sample size. Secondly, Bartlett’s test of sphericity was employed to assess the correlation between the ERSA sub-categories. The obtained *p* values of <.0001 indicated satisfactory correlation. This ensured that the data were suitable for factor analysis. Thirdly, the EFA revealed four distinct factors based on the factor loadings. In addition, CFA was conducted to confirm these four distinct factors. The fit indexes for all sub-categories demonstrated satisfactory results. It should be noted that the original study by Ambert-Dahan et al. (2018) did not include CFA, making it impossible to compare the KMO and Bartlett’s analysis values in our study.

In the present study, a significant moderate to strong correlation was observed between all sub-categories of the ERSA and all the sub-categories of the APHAB, except for the aversiveness sub-category. Particularly, the ERSA professional life sub-category exhibited the strongest positive correlation with the APHAB total score. These findings are consistent with the results reported by Ambert-Dahan et al. (2018). It is worth noting that the correlation was found to be at a moderate level in both studies. While the APHAB assesses hearing difficulties across various listening conditions, the ERSA examines the impact of hearing loss on communication capacity in different aspects of quality of life. Hence, the lack of a strong correlation may be attributed to differences in the evaluative properties of the two questionnaires. Furthermore, no correlation was found between the aversiveness sub-category of the APHAB and the ERSA sub-categories. This discrepancy can be explained by the fact that the aversiveness of the APHAB relates to individuals’ response to loud noises in close proximity, whereas the ERSA and its sub-categories measure the effects stemming from diminished perception of sounds (Ambert-Dahan et al., 2018; Cox et al., 2003).

The ERSA exhibited moderate to excellent internal consistency in both total scores and sub-category scores. Among the sub-categories assessed in our study, the ERSA/200 total score displayed the highest internal consistency (*α* = .94) while the quality-of-life sub-category showed the lowest internal consistency (*α* = .78). These findings align with those reported in the original study conducted by Ambert-Dahan et al. (2018), where the ERSA/200 total score and the quality-of-life sub-category also demonstrated the highest and lowest internal consistency coefficients, respectively (*α* = .91, *α* = .78). Furthermore, the internal consistency coefficients for the remaining sub-categories exhibited similar values in both studies. These results affirm the suitability of the Turkish version of the ERSA for use in assessing individuals with treated hearing loss, as the questionnaire demonstrates consistent internal consistency and the sub-category questions are relevant to the overall categorization.

The ERSA/150 and ERSA/200 total scores demonstrated excellent test–retest reliability with ICCs of .94 and .95, respectively. Similarly, the ICC values for the quality of life, personal life, social life, and professional life sub-categories were .90, .86, .91, and .92, respectively. These results align with the findings of Ambert-Dahan et al., who also reported excellent test–retest reliability for the ERSA/200 (ICC = .93). In their study, the ICC values for the quality of life, personal life, social life, and professional life sub-categories were .89, .85, .86, and .85, respectively (Ambert-Dahan et al., 2018). The present study demonstrates similar results to the original study, albeit with some differences. While Ambert-Dahan et al. utilized a 2-week interval for test–retest reliability and found consistent results between the first and second assessments of the ERSA, our study employed a 1-month interval. We selected a 1-month interval for the test–retest analysis because it strikes a balance. It is sufficiently short to minimize the potential impact of memory decay or external factors on participants’ responses, yet long enough to reduce the likelihood of participants recalling their initial responses. Regarding participant retention and recall bias, we encountered situations where some participants were unwilling to return for the retest due to time constraints. This led to a potential bias because those who returned for the retest systematically differed from those who did not. Therefore, certain measures were taken to encourage participants to return for the retest, including proactive communication. It is worth noting that different time intervals have been reported in the literature, emphasizing the need for standardization in future prospective studies (Marx et al., 2003). Furthermore, all correlations in the total scores and sub-categories of the ERSA were statistically significant (*p* < .001), indicating a high level of reproducibility in the measurements.

In our study, we encountered several limitations that are important to acknowledge. First, we did not collect information regarding participants’ prior familiarity with hearing loss through relatives or friends who have experienced it. This lack of data could potentially influence how participants perceive their own hearing status. Second, the use of interviews for illiterate people, especially in the presence of an interviewer and relatives, carries a risk of eliciting socially desirable responses. Participants might be inclined to provide answers that align with perceived expectations, potentially impacting the accuracy of their responses. Lastly, all participants in our study exhibited varying degrees of hearing loss. This inherent characteristic of our sample presented a challenge as it could have affected participants’ ability to hear and fully understand the interview questions. As a result, some responses may not fully reflect participants’ true experiences and perspectives.

The findings indicate that the Turkish version of the ERSA questionnaire is a reliable and valid tool with strong psychometric properties for evaluating the impact of hearing loss in individuals. The results support the feasibility of utilizing the ERSA as a means to monitor and manage the rehabilitative progress of individuals with treated hearing loss. In addition, the integration of the ERSA into standard protocols as a tool for evaluating quality of life and assessing outcomes in individuals with treated hearing loss is recommended. These findings contribute to the body of knowledge on hearing loss assessment and provide valuable insights for clinical practice and research in this field.
